# A Small-Plaque Isolate of the Zika Virus with Envelope Domain III Mutations Affect Viral Entry and Replication in Mammalian but Not Mosquito Cells

**DOI:** 10.3390/v14030480

**Published:** 2022-02-26

**Authors:** Thitigun Jaimipuk, Saranya Sachdev, Sutee Yoksan, Chutima Thepparit

**Affiliations:** Center for Vaccine Development, Institute of Molecular Biosciences, Mahidol University, 25/25 Phuttamonthon 4 Road, Salaya, Nakhon Pathom 73170, Thailand; j.thitigun@gmail.com (T.J.); saranya0918@gmail.com (S.S.); sutee.yok@mahidol.ac.th (S.Y.)

**Keywords:** Zika virus, attenuation, cell tropism, NS3, E protein, vaccine

## Abstract

An Asian Zika virus (ZIKV) isolated from a Thai patient that was serially passaged in Primary Dog Kidney (PDK) cells for attenuation displayed both big and small plaque-forming viruses by the 7th passage. Two small-plaque isolates were selected and purified for characterization as attenuated ZIKV candidates. In vitro growth kinetics showed significantly reduced titers for small-plaque isolates in Vero cells early post-infection compared to the parental ZIKV and a big-plaque isolate, but no significant difference was observed in C6/36 cells. Viral entry experiments elucidate that titer reduction likely occurred due to the diminished entry capabilities of a small-plaque isolate. Additionally, a small-plaque isolate displayed lowered neurovirulence in newborn mice compared to 100% lethality from infection with the parental ZIKV. Genomic analysis revealed the same three unique non-synonymous mutations for both small-plaque isolates: two on the envelope (E) protein at residues 310, alanine to glutamic acid (A310E), and 393, glutamic acid to lysine (E393K), and one on residue 355 of NS3, histidine to tyrosine (H355Y). Three-dimensional (3D) mapping suggests that the E protein mutations located on the receptor-binding and fusion domain III likely affect cell entry, tropism, and virulence. These ZIKV isolates and genotypic markers will be beneficial for vaccine development.

## 1. Introduction

Zika virus (ZIKV) is a mosquito-borne flavivirus belonging to the *Flaviviridae* family in the genus *Flavivirus*. This genus includes some of the most pathogenic arboviruses known to humankind, such as the dengue virus (DENV), yellow fever virus (YFV), Japanese encephalitis virus (JEV), and West Nile virus (WNV). Among these viruses, ZIKV was a relatively insignificant member of the family, with a large majority of ZIKV cases being asymptomatic and a few causing mild febrile illnesses. It was not given much attention until the outbreak in 2007 in Micronesia, which was followed by another outbreak in 2013–2014 in French Polynesia, from where ZIKV made its way East to the Americas [1]. The 2015 outbreak in Brazil is the largest ever recorded, and as of July 2019, a total of 87 countries have reported autochthonous transmission [1,2,3]. Strains causing recent outbreaks have shown increased virulence, revealing more severe effects of the infection. Microcephaly and other congenital disabilities known as congenital Zika syndrome (CZS) have been implicated in the fetuses of infected mothers [3]. Guillain–Barré syndrome (GBS)—an autoimmune disease that causes paralysis—has also been involved in ZIKV infections [4,5,6]. Recently, additional GBS and CZS cases have been reported in South and Southeast Asia, with an outbreak in Rajasthan, India in 2018 [2]. Furthermore, while the ZIKV maintains a transmission cycle through the *Aedes* spp. similarly to other flaviviruses, it has shown great capacity for direct human-to-human transmission, both sexually and through blood transfusions [7,8,9,10,11]. The virus’s broad tropism can be highlighted by its infection of human neural progenitor cells and placental cells [12,13,14,15], uterine cells [16], mice testes [17], and detection in body fluids of various kinds, including semen, vaginal secretions, and urine [18,19,20]. Its potential for re-emergence combined with unique pathogenesis warrants a further understanding of virulence factors of the virus to improve efforts in developing anti-ZIKV therapies.

Phylogenetic analysis splits ZIKV into two main geographic lineages, African and Asian [21]. Studies confirm that strains in the Asian lineage caused recent outbreaks, implicating the Asian strains to be more virulent [21,22,23]; identified genomic markers suggest an accumulation of mutations allowing the virus to infect humans to a greater degree than African strains [21,22,24]. As with all flaviviruses, ZIKV has a single-stranded positive-sense RNA genome that is ≈10.8 kb with the following components: 5′ untranslated region (5′UTR), a single open reading frame (ORF) consisting of three structural proteins (capsid (C)–premembrane/membrane (prM/M)–envelope (E)), and seven non-structural (NS) proteins (NS1–NS2A–NS2B–NS3–NS4A–NS4B–NS5), and the 3′ untranslated region (3′ UTR) [25]. Genomic analysis of viruses performed in conjunction with in vitro studies can reveal genotypic markers significant in the virulence of the virus.

An Asian strain ZIKV, CVD_06-020 (GenBank accession number MW015936), was isolated from a Thai patient with a mild fever. The virus had been undergoing serial passaging in Primary Dog Kidney (PDK) cells in attempts to attenuate the virus for developing an attenuated strain. Small-plaque morphologies were increasingly observed at the 7th passage (PDK7) amongst larger plaques. Various isolates with different plaque-size morphologies were selected for further phenotypic and genotypic characterization to gain a possible attenuated ZIKV candidate and to identify genotypic markers of the attenuated phenotype. Three non-synonymous mutations were identified in small-plaque isolates with attenuated properties, including reduced cell entry efficiency, growth kinetics in mammalian cells, and neurovirulence in mice. The lack of a distinctive change in the mosquito cell line suggests a possible effect of a mutation on receptor-binding and cell tropism. The obtained attenuated ZIKV strain and these attenuation markers will provide applicable information for developing vaccines against ZIKV and other significant flaviviruses.

## 2. Materials and Methods

### 2.1. Cells and Viruses

Vero cells (ATCC^®^ CCL-81™)—African green monkey kidney epithelial—were grown at 37 °C with 5% CO_2_ in Minimum Essential Medium (MEM) (Gibco, Grand Island, NY, USA), supplemented with 5% heat-inactivated fetal bovine serum (FBS, Gibco), and 1% penicillin/streptomycin (100 units/mL). C6/36 cells (ATCC^®^ CRL-1660™)—*Aedes albopictus* whole larvae—were cultured in Leibovitz L-15 media (Hyclone, Cramlington, UK), supplemented with 10% FBS, and 0.3% tryptose phosphate broth (TPB) and maintained at 32 °C. Primary dog kidney cells (PDK) were obtained from Hawaii-quarantined beagles. The cells were maintained in a freshly prepared MEM supplemented with 10% FBS at 37 °C without CO_2_.

Zika virus strain CVD_06-020 (GenBank Accession: MW015936) was isolated from the blood serum of a Thai patient by inoculation in the *Toxorhynchites* mosquito. The virus stock was propagated once in C6/36 and twice in Vero cells. Complete genome sequencing was analyzed to confirm the identity of the virus before serial passaging in PDK cells for virus attenuation. Briefly, PDK cells stocks were kept in −80 °C, and the individual tube could be passaged for two passages. For recovery, a tube of frozen PDK cells was thawed in the T25 cm^2^ flask. After confluent cell growth, the cells were split equally into three T25 cm^2^ flasks: two for infection and one for cell preparation for the next round of infection. Once the cells reached 80–90% confluence, two flasks were mock or virus-infected by replacing the growth media with 1 mL of serum-free media or ZIKV, respectively. After 1.5 h of adsorption, 4 mL of complete media was added to each flask, and the cells were incubated at 37 °C without CO_2_. Once cytopathic effects (CPE) of the virus-infected cells were observed, 1 mL of the growth media of the mock or virus-infected cells were inoculated into the pre-seeded PDK cells as mentioned above. Serial passages were repeatedly performed, and simultaneously, the plaque morphology and viral titer of ZIKV in each passage were determined by plaque assay.

### 2.2. Plaque Assay and Variant ZIKVs Isolation

Plaque assays were performed to determine the viral titer and corresponding plaque morphology. Vero cells were seeded on 12-well plates at a density of 3.5 × 10^6^ cells/plate one day before infection. The cells were infected with serially diluted viruses for 1 h, with 15-min rocking intervals. Post-infection, the infected cells were overlaid with semi-solidified MEM media containing 1.2% avicel (FMC Biopolymer, Philadelphia, PA, USA), supplemented with 2% FBS, and incubated at 37 °C with 5% CO_2_. Five days post-infection (dpi), cells were fixed with 4% (*v*/*v*) formaldehyde solution in phosphate-buffered saline (PBS) and stained with 1% (*w*/*v*) crystal violet.

Agarose-based plaque assays were performed to purify different plaque size-forming variants. As mentioned earlier, the traditional plaque assays were carried out, except that solidified MEM media containing 1.6% agarose (Lonza, Rockland, ME, USA) was used instead of an avicel-based media. After five days of incubation, plaques were stained with 5 mg/mL MTT (3-[4,5-dimethylthiazol-2-yl]-2,5 diphenyl tetrazolium bromide) (Applichem, Munich, Germany) in PBS solution for 3 h. Then, well-separated small and big plaques from terminal dilutions were picked and transferred to individual microcentrifuge tubes containing MEM media and incubated on a rocking plate at 4 °C overnight. The isolated viruses were propagated in pre-seeded Vero cells in 12-well plates. Virus recovery was determined by CPE observation and viral titration. Homogeneity check of virus isolates was completed using avicel-based plaque assay, and non-homogenous plaque isolates underwent a second round of purification through agarose-based plaque assay. The recovered viruses underwent a second round of propagation in C6/36 cells.

### 2.3. Zika Virus Genome Sequencing and Analysis 

Viral RNA was extracted using a High Pure Viral RNA Kit (Roche Life Science, Mannheim, Germany) following the manufacturer’s protocol. RNA was reverse transcribed with random hexanucleotides or the specific primer, Zika_R5 (Supplementary Table S1) using ImpromII^TM^ reverse transcriptase kit (Promega, Madison, WI, USA). The transcribed cDNA was used as a template to amplify a set of fragments covering the whole ZIKV genome using specific primers (Supplementary Table S1) with either Phusion-High Fidelity (New England BioLabs, Ipswich, MA, USA) or Platinum SuperFi DNA polymerase enzyme (Invitrogen, Carlsbad, CA, USA). The PCR products were purified using Gel/PCR DNA fragments excision kit (Geneaid, Taipei, Taiwan), following the manufacturer’s protocol. The purified DNA fragments were sent to either Macrogen (Seoul, Korea) or 1st BASE Apical Scientific (Singapore) for nucleotide sequencing. The nucleotide sequences were analyzed and assembled using the BioEdit Sequence Alignment Editor Program [26]. The nucleotide and the translated amino acid sequences were aligned with the parental sequences to identify mutations. The nucleotide sequences of ZIKVs isolates were analyzed periodically to ensure the genotypic stability of the viruses.

### 2.4. Structural Analysis of Proteins with Mutations

Three modeling programs were used to perform structural analysis of mutations on respective proteins. Mutations identified by genomic analysis were three-dimensionally mapped onto proteins obtained from the Protein Data Bank (PDB) using PyMol [27]. Dynamut [28] was used to visualize and analyze the potential impact of mutations on protein dynamics and stability resulting from vibrational entropy changes. Interactomics of the mutational residue was also visualized with surrounding residues. Each mutation was individually analyzed. The SWISS-Model [29] was used to visualize and assess changes in the polarity and charge of the single point mutations on each protein.

### 2.5. Growth Kinetics Studies

Vero and C6/36 cells were seeded on 6-well plates one day prior to infection. The pre-seeded cells with 70–80% confluence were infected with ZIKV-CVD_06-020, ZIKV-PDK7-B5, ZIKV-PDK7-S3, or ZIKV-PDK7-S1.4 at a multiplicity of infection (MOI) of 0.01. Viruses were incubated for 1 h at 37 °C for Vero or 32 °C for C6/36 cells with rocking intervals of 10 min to allow virus adsorptions. After infection, the inoculum was removed. The cells were treated with acid glycine to inactivate extracellular viruses, which was followed by three PBS washes, after which a pre-warmed growth medium was added to the cells. Small aliquots of media were periodically recovered for plaque assay titration on Vero cells. Growth kinetic curves were constructed on Microsoft Excel and statistically analyzed using two-way ANOVA. The growth kinetics were performed in triplicates with duplicate titration of each collected sample. A mean titer of each time point was plotted with error bars representing the standard deviation. A significant difference is achieved at *p*-value ≤ 0.05.

### 2.6. Viral Entry Assays

The pre-grown Vero and C6/36 cells in 24-well plates with 80–90% confluence were washed with PBS before infection with ZIKV-CVD_06-020, ZIKV-PDK7-B5, or ZIKV-PDK7-S1.4 at MOI of 10. The cells were incubated with viruses for 1.5 h at 37 °C for Vero cells and 32 °C for C6/36 cells with rocking intervals of 10 min. After virus removal, the cells were treated with acid glycine for 1 min to inactivate the remaining un-internalized viruses and washed 3 times with PBS before adding the complete medium. The infected cells were cultured for 24 h. Then, the supernatant was removed, and the cells were washed with PBS before adding TRI Reagent (MRC Inc., Cincinnati, OH, USA) for RNA extraction following the manufacturer’s instructions. Briefly, 100 µL of TRI Reagent was added to each well. The cells were homogenized by pipetting and then transferred to a tube. The homogenate was stored at room temperature for 5 min before adding 20 µL of chloroform (BBH, Poole, Dorchester, Dorset, UK) followed by vigorous mixing with a vortex. After incubation at room temperature for 10 min, the mixture was centrifuged at 12,000× *g* for 15 min. Then, the upper phase was transferred to a new tube, a 1:1 ratio of isopropanol (Merck, Darmstadt, Germany) was added, and the mixture was incubated for 10 min at room temperature. After centrifugation at 12,000× *g* for 10 min, the resulting pellet was washed with 75% ethanol (Merck, Darmstadt, Germany). Then, the ethanol was removed by centrifugation at 7500× *g* for 5 min. The pellet was dried and dissolved in 20 µL of pre-warmed DEPC water. RNA was subjected to one-step real-time qRT-PCR using a KAPA Probe Fast Universal One-step qRT-PCR (Kapa Biosystem, Inc., Wilmington, MA, USA). The in vitro transcribed RNA containing ZIKV_prME, *A. aegypti*_RpS17, and Mus_18sRNA genes was used as a template to generate a standard curve. Probes and primers are shown in Supplementary Table S2. The reaction mixers were composed of a 1 x KAPA Probe Fast qPCR Master mix, 0.2 μM of each primer, 0.2 μM of a fluorogenic probe, and RNase-free water in a final volume of 20 μL. The PCR condition is as follows: 42 °C for 5 min, 95 °C for 2 min following 40 cycles of 95 °C for 30 s and 60 °C for 20 s. The RNA copies were calculated according to the standard curve. A single cell of Vero contains four hundred copies of 18sRNA genes, and one C6/36 cell contains four RpS17 copies. The viral copy number per cell for each virus was determined and statistically compared among each virus group using one-way ANOVA. The bar graph represents the mean with SD. A significant difference is achieved at *p*-value ≤ 0.05.

### 2.7. Mouse Neurovirulence

Pregnant ICR (Institute of Cancer Research) mice, a strain of albino mice originating from a Swiss mouse colony, were obtained from Nomura Siam International (Thailand). Two-day-old ICR mice were intracranially injected (i.c.) with 2 × 10^4^ pfu/mouse of normal saline (NSS) (n = 10), ZIKV-CVD_06-020 (n = 15), or ZIKV-PDK7-S1.4 (n = 13). The injected mice were observed for 21 days to determine morbidity and mortality. The survival curve was constructed and analyzed using Log-rank (Mantel-Cox) tests from GraphPad Prism version 9.0.2 (GraphPad Software, San Diego, CA, USA). A significant difference is achieved at a *p*-value ≤ 0.0001.

## 3. Results

### 3.1. Isolation of Plaque Size Variants from a Serially Passaged ZIKV in Primary Dog Kidney Cells

**Figure 1:**
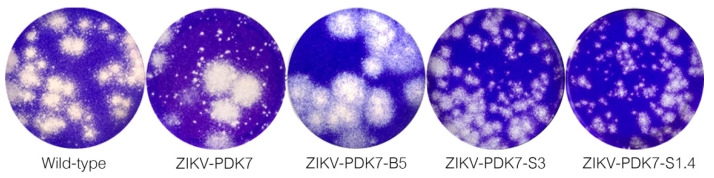
Plaque morphology of wild-type, ZIKV-PDK7, ZIKV-PDK7-B5, ZIKV-PDK7-S3, and ZIKV-PDK7-S1.4 variants. Plaque morphology of ZIKV variants were observed by avicel-based plaque assays in Vero cells at 5 dpi. ZIKV-CVD_06-020, an Asian wild-type ZIKV, contains mixed plaque variants with plaques diameter ranging from 0.1 to 0.5 mm. ZIKV-PDK7—a wild-type ZIKV serially passaged in PDK cells for seven passages—shows an increase in small-plaque-forming virus population. ZIKV-PDK7-B5—a big plaque isolated from ZIKV-PDK7—presented homogeneous big-plaque-forming viruses with diameter ≥ 0.4 mm. ZIKV-PDK7-S3 and ZIKVV-PDK7-S1.4—small-plaque variants purified from ZIKV-PDK7—showed major populations of small-plaque-forming viruses with diameter < 0.2 mm.

An Asian ZIKV virus strain CVD_06-020 (wild-type and parental ZIKV) was serially passaged in PDK cells in an attempt to attenuate the virus. At the 7th passage, where the virus was named ZIKV-PDK7, small plaques (diameter < 0.2 mm) representing an attenuated phenotype were observed in high frequency (Figure 1). Variants were isolated through agarose plaque assay on Vero cells to obtain attenuated virus candidates and investigate the genotypic determinants of small-plaque forming variants. The big-plaque isolate (diameter ≥ 0.4 mm) was obtained to ensure that any genotypic markers found were not characteristic of all ZIKV-PDK7 variants. Five big and five small plaques were initially picked and purified. The most homogenous big-, ZIKV-PDK7-B5 (Big plaque no.5), and small-, ZIKV-PDK7-S3 (Small plaque no.3), plaque isolates were then selected (Figure 1). A second small plaque isolate, ZIKV-PDK7-S1, underwent another round of plaque purification to obtain a more homogenous population, named ZIKV-PDK7-S1.4 (Figure 1). The three selected plaques were further propagated in C6/36 cells for volume amplification.

### 3.2. Two Small-Plaque ZIKV Isolates Possess Three Unique Non-Synonymous Mutations

**Figure 2:**
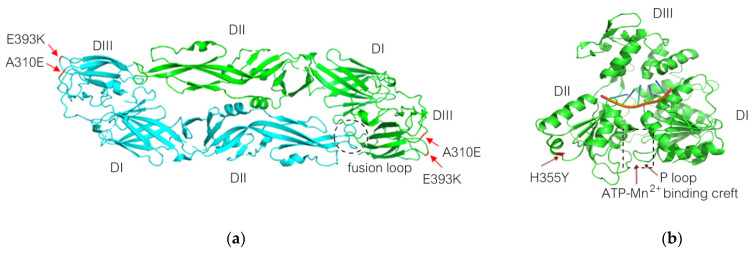
(**a**) Two E mutations, A310E and E393K of ZIKV-PDK7-S3 and ZIKV-PDK7-S1.4, are mapped on the ZIKV E protein dimer using PBD: 5LBV as a template. Both mutations are located at domain III (DIII) of the E protein as red indicates. (**b**) NS3 mutation H355Y of ZIKV-PDK7-S3 and ZIKV-PDK7-S1.4 is mapped onto the ZIKV NS3 protein using PBD: 5GJB as a template. The mutation is located at domain II (DII) of the helicase–RNA complex. The dotted square presents an ATP/Mn2+ binding site in a cleft of domain I and II (DI and DII), which is composed of residues G197, K200, T201, R202 (P loop), E286, N330, R459, and R462.

**Figure 3:**
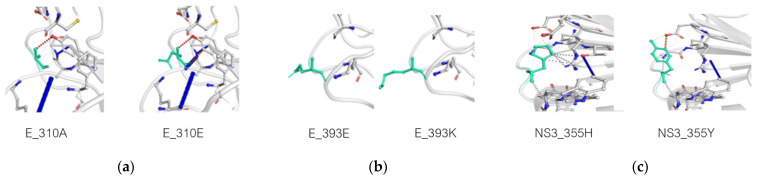
Effects of mutation on E and NS3 helicase protein stability and interactomics in the mutation region. Dynamut normal mode analysis (EnCoM) was used to analyze and visualize the potential impact of the mutations dynamics and stability resulting from vibrational entropy changes. PBD: 5LBV was used as the model of E protein. A310E (**a**) and E393K (**b**) are predicted to be stabilizing and decrease the flexibility of the protein. PBD: 5GJB was used as the model of NS3 helicase protein. The H355Y mutation is predicted to be a slightly destabilizing mutation and slightly increases the flexibility of the protein (**c**). Change in interactomics of the mutation residue (shown in green) with surrounding residues was also predicted for A310E (**a**), E393K (**b**), and H355Y (**c**).

**Figure 4:**
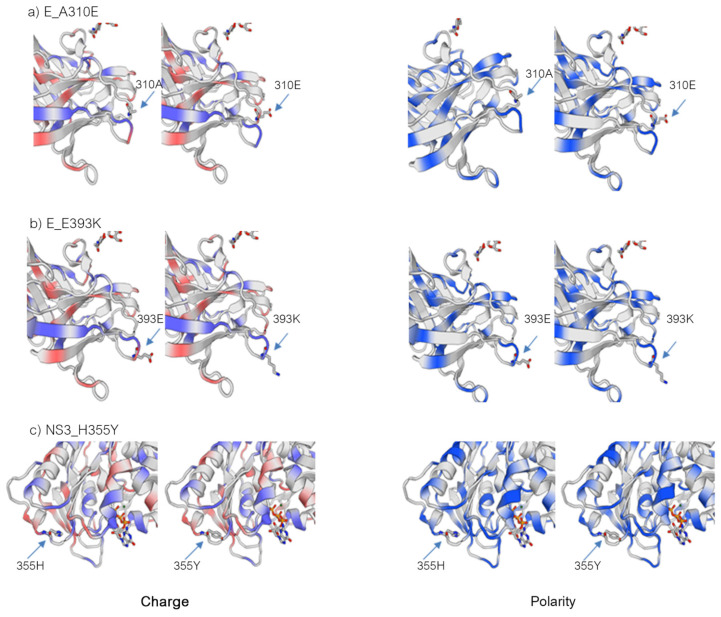
Effects of mutations on polarity and charge of protein in the mutation region. SWISS-MODEL was used to model and visualize the changes in charge (**left**) and polarity (**right**) in the mutation residues of E:DIII and NS3:DII, using PBD: 5LBV and PBD: 5GJB, respectively. The A310E mutation on E:DIII (**a**) shows a change from an uncharged, nonpolar residue to a negatively charged, polar residue, while the E393K mutation (**b**) goes from a negatively charged, polar residue to a positively charged polar residue. The H355Y mutation on NS3 (**c**) shows a change from a positively charged, polar residue at pH 7.2 to an uncharged, polar residue.

Complete genome analysis of the two small-plaque isolates compared to the wild-type ZIKV and the big plaque isolate revealed three unique non-synonymous mutations that resulted in amino acid changes in the envelope (E) and helicase (NS3) proteins. The first mutation was located on position 600 of the polyprotein or position 310 of the E protein, resulting in an amino acid change from alanine (A) to glutamic acid (E) (A310E). A second point mutation in the E protein presented at position 683 of the polyprotein or position 393 of the E protein, from glutamic acid (E) to lysine (K) (E393K). The third mutation was on position 1857 of the polyprotein or 355 of the NS3, resulting in an amino acid change from histidine (H) to tyrosine (Y) (H355Y) (Table 1). In contrast, those residues in the big-plaque isolate, ZIKV-PDK7-B5, retained precisely the same amino acids as found in the wild-type ZIKV (Supplementary Figure S1). Three-dimensional (3D) mapping of the mutations demonstrated that the E protein mutations are both located on the outer region of domain III (EDIII) (on the receptor-binding and fusion domain) and are close to one another (Figure 2a). The NS3 mutation was on the outer part of domain II (NS3DII) (Figure 2b). Dynamut and SWISS-MODEL were used to predict the effects of the residue changes on protein dynamics. The values of change in energy between wild type and variants (ΔΔG) and a change in vibrational entropy energy (ΔΔSVib) are shown in Supplementary Table S3. The A310E mutation and E393K mutations were both found to be stabilizing and decreased the flexibility of the protein. There were also changes in interactions with surrounding residues (Figure 3a,b). Of note, the A310E mutation is a change from an uncharged, nonpolar alanine to a negatively charged, polar glutamic acid, while the E393K mutation is a change from a negatively charged, polar glutamic acid to a positively charged polar lysine (Figure 4a,b). The H355Y NS3DII mutation was also a stabilizing mutation, but it slightly increased the flexibility of the protein specifically at the mutation point with noticeable changes in interactions with surrounding residues (Figure 3c), and it was a change from a positively charged, polar histidine to an uncharged, polar tyrosine (Figure 4c). As EDIII is involved in viral entry into host cells, and NS3 is involved in replication, the next step was to see if these mutations affected growth kinetics in cell lines.

### 3.3. The Small-Plaque ZIKV Isolates Had Delayed Start to Replication in Mammalian but Not Mosquito Cells

**Figure 5:**
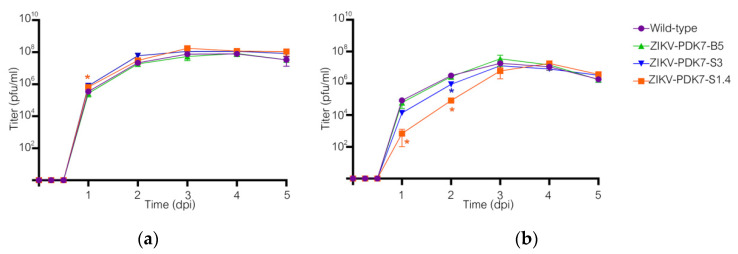
Growth kinetics of wild-type and ZIKV variants. Viral growth kinetics were determined in C6/36 (**a**) and Vero (**b**) cells. The cells were infected with ZIKVs; wild-type (circle), ZIKV-PDK7-B5 (triangle), ZIKV-PDK7-S3 (inverted triangle), and ZIKV-PDK7-S1.4 (square) at an MOI of 0.01. The experiment was performed in triplicate with duplicate plaque counting. The mean viral titers at each time-point were plotted with the error bar representing the standard deviation (SD). Two-way ANOVA was used to statistically analyze the data against the wild-type strain. Asterisks (*) indicate a statistically significant difference (*p* < 0.05). If not displayed, the data did not show significant differences.

**Figure 6:**
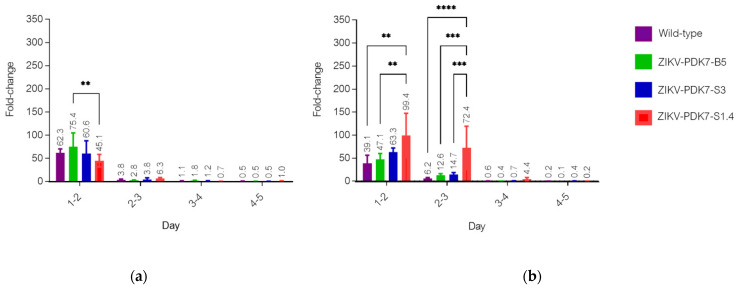
Ratios of replication rate per day of ZIKV isolates. The fold-changes of replication rate were calculated from viral growth kinetics in C6/36 (**a**) and Vero cells (**b**). Bar graphs represent the ratio of increasing titers of ZIKV isolates per day between day 1 and day 5, and the error bars represent the SD. Fold-changes of viral yield per day are shown in the figure. A significant difference is calculated by using two-way ANOVA with Tukey’s multiple comparisons test. *p*-values: 0.1234 = ns; 0.0021 **; <0.0002 ***; <0.0001 ****.

The replication efficiency of various ZIKV isolates was determined in vitro by growth kinetics assay on Vero and C6/36 cells as mammalian host and vector models, respectively. While there is a slight difference in the viral production of ZIKV variants in C6/36 cells throughout the 5 dpi (Figure 5a), in Vero cells, small-plaque variants, especially ZIKV-PDK7-S1.4, had significantly reduced viral yields by approximately two log10 at early dpi compared to the wild-type and the big-plaque variant ZIKV-PDK7-B5 (Figure 5b). However, at three dpi, the viral titer of the ZIKV-PDK7-S1.4 isolate accelerated to titers comparable to other strains, which could correspond to an enhanced replication rate per day of the ZIKV-PDK7-S1.4 isolate. Fold-change per day of the viral titer was calculated for all days. On 1 dpi, virus titers were 8.3 × 10^5^ pfu/mL for the wild-type, 6.1 × 10^5^ pfu/mL for ZIKV-PDK7-B5, and 6.8 × 10^2^ pfu/mL for ZIKV-PDK7-S1.4. However, between days 1 and 2 dpi, ZIKV-PDK7-S1.4 titer increased 99-fold, while wild-type and ZIKV-PDK7-B5 titers increased only 39-fold and 47-fold, respectively. Similarly, between days 2 and 3 dpi, ZIKV-PDK7-S1.4 titer increased 72-fold, while wild-type and ZIKV-PDK7-B5 titers increased only 6-fold and 13-fold, respectively (Figure 6b). Notably, the ZIKV-PDK7-S3 virus may not be a homogeneous isolate suggested by the sequence variation shown in the chromatography (Supplementary Figure S1). This could be why the viral growth kinetics (Figure 5b) is similar to the parent strain.

### 3.4. Delayed Start to Replication of the Small-Plaque Isolate in Vero Cells Is Due to Diminished Capability in Cell Entry

**Figure 7:**
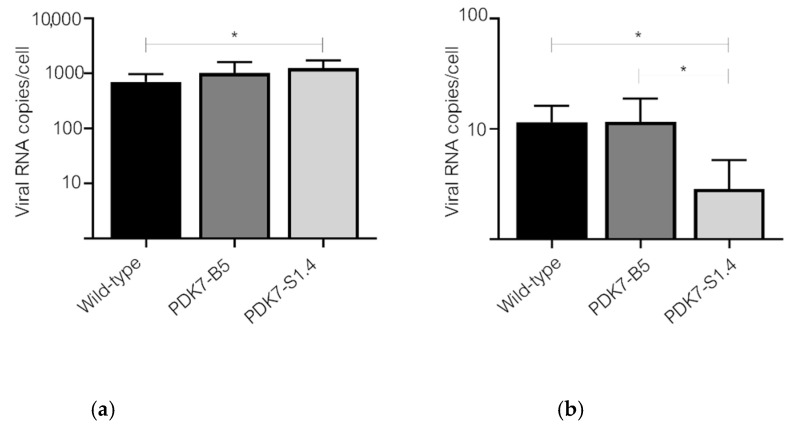
Viral entry into C6/36 (**a**) and Vero (**b**) cells was determined by real-time PCR. The viral RNA copies number per cell was determined by real-time PCR. The cells were infected with ZIKVs, wild-type, ZIKV-PDK7-B5, and ZIKV-PDK7-S1.4, at an MOI of 10. After viral adsorption, the viruses were removed and treated with acid glycine to inactivate the remaining un-internalized viruses. After 24 h post-infection, the infected cells were harvested for RNA extraction and subjected to one-step real-time PCR. The experiment was performed in triplicate with triplicate real-time PCR quantification. The bar graph represents mean values, with the error bar representing SD. One-way ANOVA was used, and asterisks (*) indicate a statistically significant difference (*p* < 0.05).

The two small-plaque isolates ZIKV-PDK7-S3 and ZIKV-PDK7-S1.4 contain the same three unique non-synonymous mutations in E and NS3 proteins. We hypothesized that the two mutations on the E protein (A310E and E393K) affect cell receptor binding and subsequently viral entry into Vero but not C6/36 cells. Therefore, viral entry into the C6/36 and Vero cells of each isolate was determined after 24 dpi by real-time PCR to examine the viral copies per cell of each ZIKVs infected group. The results showed a significant reduction in the small-plaque variant, ZIKV-PDK7-S1.4, entry into Vero but not C6/36 cells, compared to the parental ZIKV and the big-plaque isolate (Figure 7a,b), whereas the degree of entry of the big-plaque isolate was comparable to the wild-type ZIKV.

### 3.5. The Small-Plaque Variant Had Reduced Virulence in Newborn Mice

**Figure 8:**
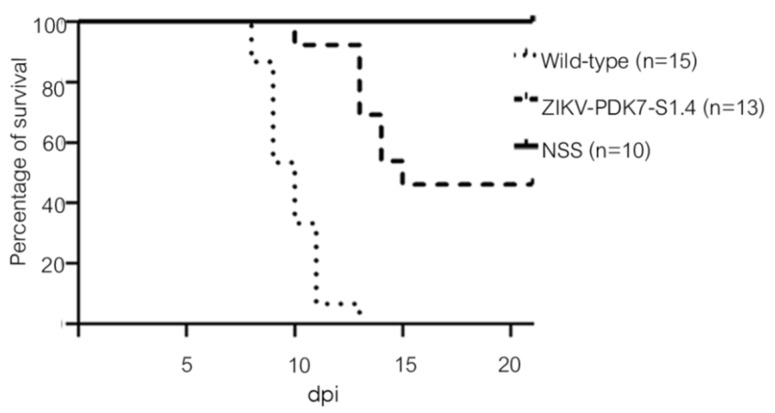
Mouse neurovirulence. Two-day-old ICR mice were intracranially injected with 2 × 10^4^ pfu of the wild-type ZIKV (dotted line), ZIKV-PDK7-S1.4 (dash line), or NSS (solid line). The mice were observed daily for morbidity and mortality for 21 days. The survival rate analyzed by Log-rank (Mantel–Cox) tests shows significant differences between the mice infected with the ZIKV-PDK7-S1.4 isolate and the wild-type ZIKV infection (*p* < 0.0001).

The neurovirulence of the ZIKV variants was determined in newborn mice. We intracranially injected newborn ICR mice with either normal saline solution (NSS), wild-type ZIKV, or the small-plaque ZIKV-PDK7-S1.4 isolate and monitored them daily for morbidity and mortality. The wild-type ZIKV infected mice showed neurological symptoms including tremors, ataxia, and paresis on day 8, while those symptoms were observed in the ZIKV-PDK7-S1.4 injected mice on day 10. With these signs, most of the mice developed paralysis or death. However, three ZIKV-PDK7-S1.4-injected mice could be recovered. Some severe cases displayed paralysis or seizure before death. By 13 dpi, all 15 mice infected with the wild-type ZIKV died. In comparison, 46% of the ZIKV-PDK7-S1.4-infected mice (6/13) survived until 21 dpi (Figure 8). All the surviving mice were healthy or had no symptoms throughout the experiment. The results indicate lower neurovirulence in newborn mice upon ZIKV-PDK7-S1.4 infection.

## 4. Discussion

Three ZIKV isolates with different plaque sizes were successfully recovered from the ZIKV-PDK7, the Asian ZIKV strain CVD_06-020 serially passaged in PDK cells for seven passages. The two small-plaque isolates—ZIKV-PDK7-S3 and ZIKV-PDK7-S1.4—shared the same three non-synonymous mutations; A310E and E393K in E protein, and H355Y in NS3 protein. The big-plaque isolate ZIKV-PDK7-B5 presented the same genotype as the parental strain, suggesting that at least one mutation contributes to small plaque morphology. Upon growth kinetics study, the small-plaque variant, ZIKV-PDK7-S1.4, initially showed significant viral yield reduction, but the replication accelerated to comparable production as the wild-type ZIKV in mammalian but not in C6/36 cell lines. The varying effect on different cell types suggests that at least one of the mutations found in the small-plaque variant could potentially have various effects on pathogenesis and virulence in different hosts, as also observed in the newborn ICR mice. To determine which specific mutations contribute to which effect, a reverse genetics study should further be employed. However, reviewing these mutations and understanding their functional analysis can still elucidate essential information.

The E protein mutations, A310E and E393K, are located close to each other on the outer region of domain III (EDIII) (Figure 2a). EDIII plays a crucial role in host–virus receptor recognition and envelope fusion [30]. Amino acid variations on the EDIII have affected host cell tropism and virulence [30,31]. Multiple studies have reported the significance of the E393 residue, including how it is a critical difference between African strains that have aspartic acid (D) and Asian strains that have glutamic acid (E) on this position [32,33,34]. It is located in the domain’s lateral bridge (LR) region, which is essential in viral fusion with host cells. The E393 residue has also been implicated in EDIII binding affinities to receptors and antibodies [33,34]. Some other crucial residues in the E protein have been reported, including residue E309, the essential interacting residue with the anti-EDIII-LR antibody [34], and residues V317I and D393E (V603I and D679E of the polyprotein) were found to be viral virulence determinants [35]. Therefore, this E protein mutation, E393K, of small-plaque isolates could reduce viral virulence. Due to proximity, it is likely that the E310 residue mutation found in this study could also affect functions of EDIII as E393 does, albeit to a lesser extent. The A310E mutation changes the uncharged, nonpolar alanine to a negatively charged, polar glutamic acid, while the E393K changes the negatively charged, polar glutamic acid to a positively charged, polar lysine (Figure 3). Furthermore, the mutations predictably affected the interactomics of the mutant residue with surrounding residues (Figure 4). Taken together, the mutation at the two residues may cause conformational, but more importantly, biochemical changes on the E protein, affecting protein–receptor interaction and viral–host membrane fusion resulting in cell tropism alteration. There are 90 E protein homodimers on each Zika virion resulting in 180 receptor binding sites on EDIII [36]. A mutation at the genome level would effectively alter all these receptor binding sites and significantly affect the virus’s affinity and avidity toward cell receptors. We reported that the delayed viral production of small-plaque isolates resulted from the inefficiency of cell entry in mammalian but not mosquito cells. The results implied that the alteration of these E protein residues specifically affected Vero cell receptor-binding but not C6/36 cells. A deep mutational scan study of the ZIKV envelope protein also highlights the existence of mammalian- or mosquito-specific mutations on EDIII that affect viral entry and fusion into cells [31]. The distinct effect of a particular mutation on diverse cell types was also demonstrated in a membrane (M) protein mutation, which caused a reduction in viral replication in Vero but not C6/36 cells [37].

Another possible effector of viral production profile of the small-plaque variant is the role of the mutation at residue 355 of domain II of the NS3 helicase protein (NS3DII), H355Y. The helicase protein plays an active role during RNA replication within host cells. While the 355 residue has not been identified in any binding sites or motifs, its location is close to the DI-DII ATP-Mn^2+^ binding cleft, which produces energy from ATP hydrolysis to unwind the RNA duplex (Figure 2b). This region is also near the motor regions of DII that rotate upon binding to RNA [38,39]. Furthermore, the amino acid change from a positively charged histidine to an uncharged tyrosine could affect the folding or interactomics of the region with neighboring residues (Figure 3). The impact of H355Y mutation was analyzed using the Dynamut normal mode analysis (EnCoM) program, and it is predicted to be a slightly destabilizing mutation and slightly increases the flexibility of the protein. While this functional analysis is speculative, this specific mutation of H355Y has previously been reported to exist in circulation. The mutation was first identified in Haiti and is not observed in any pre-epidemic Asian strains or the French Polynesian strain. Since its identification in Haiti, it has also been reported in epidemic South American strains such as in Brazil and Venezuela. While the significance of this mutation is not yet identified, it has been suggested to be an epidemic-related mutation [32,40,41]. The occurrence of this mutation in the two small plaque isolates could potentially contribute to increased replication of the virus within mammalian cells. Upon analysis of the ZIKV-PDK7-S1.4 growth kinetics in Vero cells, it can be seen that while the titer on day one is significantly less, the increasing rate of titer per day is higher than the parental strain, which is most evidently observed at 2–3 dpi (Figure 6). The lower viral yields of the ZIKV-PDK7-S1.4 during early post-infection might result from the inefficiency in cell entry of the small-plaque virus due to E protein mutations, whereas enhanced replication rate might be a result of the NS3 mutation. Interestingly, the other small-plaque isolate, ZIKV-PDK7-S3, which shares the same three mutations with ZIKV-PDK7-S1.4, seems not to show the same delayed phenotype in Vero cells. This phenotype might be due to the higher heterogeneity amongst the ZIKV-PDK7-S3 isolate, which had a higher proportion of big plaque-forming units (Figure 1). The chromatography of the ZIKV-PDK7-S3 isolate (Supplementary Figure S1) also showed more sequence variation suggesting less homogeneity, which may contribute to the higher titer in growth kinetics as compared to ZIKV-PDK7-S1.4.

In addition, the ZIKV-CVD_06-020 genome was analyzed for virulence markers against previous studies. The amino acids identified to affect ZIKV virulence are summarized in Table 2. Our wild-type ZIKV strain contains largely non-virulent amino acids residues but caused mortality in newborn mice. Meanwhile, ZIKV-PDK7-S1.4 showed reduced neurovirulence in newborn mice and can be used to develop an attenuated ZIKV strain. The study of ZIKV genetics reveals many mutations that could affect mouse neurovirulence, such as the mutation of glycosylation of E protein, N154Q, which caused decrease neurovirulence in A129 mice and strongly induced neutralization antibodies [42,43], while the flexible hinge region (residue 273–277) and glycosylation at 154 of E protein were identified as attenuation markers of Murray Valley encephalitis virus and showed less neuroinvasiveness and delayed time to death in mice [44]. Therefore, mutations in ZIKV-PDK7-S1.4, especially in E protein, might cause attenuation in newborn mice, correlating to the results in mammalian cells.

Small-plaque variants are typically expected to have delayed replication, but the possibility of counteracting mutations can exist. This study highlights the importance of residue E393 and introduces E310 as a potential genotypic marker for not only small plaque phenotypes but also receptor interactions and entry. Studies should further investigate the specific effect of the novel glutamic acid to lysine change, as most previous studies have only analyzed the impact of the D393E change from African to Asian strains. Furthermore, the random occurrence of the H355Y mutation on NS3 that has been found in epidemic strains warrants further study into the significance of this mutation as well. This study further fuels the importance of EDIII residues and, more importantly, displays the varying effects of the same mutation on different systems.

In conclusion, these mutations of small-plaque variants could have potential relevance in identifying key genotypic markers that affect virulence in viral engineering for therapies. As we obtain a deeper understanding of ZIKV functional genomics, studying the effects of individual mutations, not just those in this study, could be the key to tackling the diverse virulence and seemingly sporadic severe cases of ZIKV.

## 5. Conclusions

ZIKV-PDK7, a wild-type ZIKV (strain CVD06_020) passaged in PDK cells for seven passages, generated a potential attenuated virus with a small-plaque size in a mixed population. We isolated one big and two small-plaque variants from the ZIKV-PDK7. The big-plaque isolate showed the same phenotype and genotype as the wild-type ZIKV. A small-plaque ZIKV variant, ZIKV-PDK7-S1.4, had significantly delayed replication in Vero cells, resulting from inefficient cell entry of the virus. In contrast, there was no significant change in C6/36 cells. The small-plaque isolate had reduced neurovirulence in newborn mice compared to the parental ZIKV strain. Genetic analysis of the small-plaque variants revealed three unique non-synonymous mutations at E domain III (A310E, E393K), which is responsible for viral entry and fusion, and the NS3 domain II (H355Y), which is involved in viral replication. These three mutation residues might be potential attenuation markers affecting virulence, viral pathogenesis, and cell tropism. These key genotypic markers need to be functionally elucidated and could potentially serve for further applicable vaccine development.

## Figures and Tables

**Table 1:** Non-synonymous mutations of the ZIKV-PDK7-S3 and ZIKV-PDK7-S1.4 isolates.

Nucleotide ^1^	Amino Acid ^2^	Region on ZIKV
C1906A	A600/310E	Envelope
G2154A	E683/393K	Envelope
C5676T	H1857/355Y	NS3

^1^ The nucleotide of the wild-type ZIKV-CVD_06-020 at the mutated position in the ZIKV genome followed by the mutated nucleotide in the small-plaque variants. ^2^ The amino acid of the wild-type ZIKV-CVD_06-020 at the mutated position in the polyprotein/the position in the viral protein and the mutated amino acid in the small-plaque variants.

**Table 2:** Summary of amino acid mutations increasing virulence in infected mice.

Amino Acid Mutation ^1^	Region	ZIKV-CVD_06-020	References
154N	E protein domain I/glycosylation site	N	[42]
V473M	E protein/ trans membrane helix	V	[45]
V603I, D679E *	E protein domain III	I, E	[35]
S139N *	pr domain	S	[21]
A117V	NS2A	A	[46]

^1^ Number with asterisk (*) indicates the position of mutated amino acid in the polyprotein. If not displayed, the numbers are the positions in the specified viral proteins.

## Data Availability

The complete genome sequence of ZIKV-CVD_06-020 has been deposited in the GenBank database under accession number MW015936 (https://www.ncbi.nlm.nih.gov/nuccore/MW015936, accessed on 22 November 2021). The Protein structures used for 3-D mapping analysis in this study are retrieved from the Protein Data Bank under IDs 5LBV (https://www.rcsb.org/structure/5LBV, accessed on 14 November 2021) and 5GJB (https://www.rcsb.org/structure/5GJB, accessed on 14 November 2021).

## Supplementary Materials

The following supporting information can be downloaded at: https://www.mdpi.com/article/10.3390/v14030480/s1, Figure S1: Chromatograms of mutations, Table S1: Primers used for whole-genome ZIKV sequencing, Table S2: Primers and probes for real-time PCR, and Table S3: Energy and vibrational entropy changes upon the mutations.
