# Supplementary material for: A Small-Plaque Isolate of the Zika Virus with Envelope Domain III Mutations Affect Viral Entry and Replication in Mammalian but Not Mosquito Cells
Source: Viruses. 2022 Feb 26;14(3):480. doi: 10.3390/v14030480 (PMC8954177; doi:10.3390/v14030480)
Supplement: Supplementary file 1 [file viruses-14-00480-s001.zip › viruses-1534881-supplementary.pdf]

Supplementary Materials

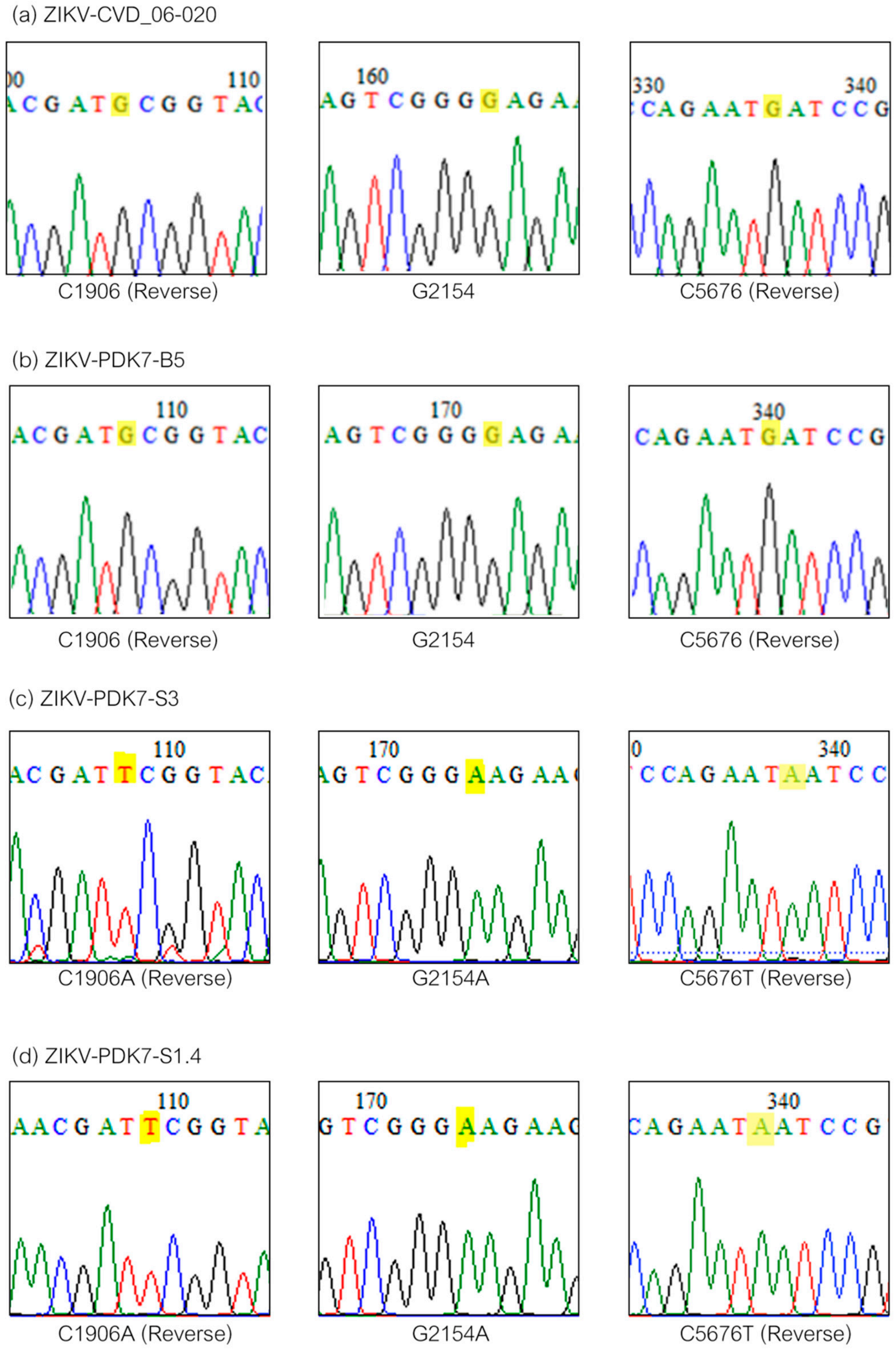

Figure S1. Chromatograms of mutations **a)** ZIKV-CVD_06-020 or wild-type, **b)**ZIKV-PDK7-B5, **c)** ZIKV-PDK7-S3, and **d)** ZIKV-PDK7-S1.4. The nucleotide positions at 1906, 2154, and 5676 represent the mutation points A310E, E393K, in E protein and H355Y in NS3, respectively. "(Reverse)" indicates that a reverse primer was used to read that sequence portion

Table S1. Primers for whole genome ZIKV sequencing

Region	Primer name	Primer sequence (5′-3′)
5′UTR-E	ZIKV_F1	AGTTGTTGATCTGTGTGAATCAGACTGC [1,2]
	ZIKV_R213	CCATGACCCAGCAGAAGTCC[1]
	ZIKV_F774	TCCACTAGGAAGCTGCAAA[1]
	ZIKV_F1084	GAGATGGATGGTGCAAAGGGAAG[1]
	ZIKV_R1_2040	CTGGGGTCAGAGTTTGCATG[1,2]
E-NS1	ZIKV_F2_1957	CAGTGGAGGTACAGTACGCAGG[1, 2]
	ZIKV_NS1F	GATGTGGGGTGCTCGGTGGACT[1]
	ZIKV_R2_4123	TTCTTCACACTGCCTTTCCCC [1,2]
NS1-NS3	ZIKV_F3_3978	CCACGCACTGACAAYATCACCTTGG [1,2]
	ZIKV_NS3F	AGTGGTGCTCTATGGGATGTG[1]
	ZIKV_R3_6040	CCACCTCCATACAGATACTCATCTC [1,2]
NS3-NS5	ZIKV_F4_5942	TGGACCCATGCCTGTCACAC[1, 2]
	ZIKV_F5886	AGATTCCAGGAGATGCCTAAAG[1]
	ZIKV_NS4AF	GGAGCGGCTTTTGGAGTGATGG[1]
	ZIKV_NS4BF	AATGAACTCGGATGGTTGGAG[1]
	ZIKV_F7418	AAAGATGGGACAGGTGCTACTC[1]
	ZIKV_R4_9037	CCCATCATGTTGTACACACAGCTC [1,2]
NS5-5′UTR	ZIKV_F5_8800	CGTAGCAATGCAGCATTAGGGG [1,2]
	ZIKV_NS5R	GAAGGGTCTACACCTGGAGTGCTA[1]
	ZIKV_F10117	GAGTGTGGATTGAGGAGAACGAC[1]
	ZIKV_R5	AGACCCATGGATTTCCCCAC [1,2,3]

[1] Primers for sequencing

[2]Primers for both amplification and sequencing of each fragment

[3] Primer for cDNA synthesis

Table S2. Primers and probes for real-time PCR

	Detection	Primer and probe	Primer and probe sequences (5′-3′)
Virus	Zika envelope	ZIKV_BonE_F*	AGYCGYTGYCCAACACAAG
		ZIKV_BonE_R*	CACCARRCTCCCYTTGCCA
		ZIKV_BonE probe*	FAM-CCTMCCTYGAYAAGCARTCAGACACYCAA-BHQ1
Housekeeping	40SRibosomal Protein S17 *A. aegypti*	RPS17_F**	ACATCTGATGAAGCGCCTGC
		RPS17_R**	ACACTTCCGGCACGTAGTTGT
		RPS17_probe**	JOE-CACTCCCAGGTCCGTGGTATCTCCATC-BHQ1
	MusM_18srRNA	MusM_18srRNA_F	GTAACCCGT TGAACCCCATT
		MusM_18srRNA_R	CCATCCAATCGGTAGTAGCG
		MusM_18srRNA_probe	JOE-TCCCAGTAAGTGCGGGTCATAAGC–BHQ1

*Corman VM, Rasche A, Baronti C, et al. Assay optimization for molecular detection of Zika virus. Bull World Health Organ. 2016;94(12):880-892. doi:10.2471/BLT.16.175950

**Cox J, Brown HE, Rico-Hesse R. Variation in vector competence for dengue viruses does not depend on mosquito midgut binding affinity. PLoS Negl Trop Dis. 2011;5(5):e1172. doi:10.1371/journal.pntd.0001172

Table S3. Energy and vibrational entropy changes

Region	Mutations[1]	ΔΔG (kcal.mol-1.K-1)[2]	ΔΔSVib (kcal.mol-1.K-1)[3]
Envelope	A310E	1.861	-2.326
	E393K	1.629	-2.036
NS3	H355Y	-0.059	0.073

1 Mutations of small-plaque isolates

2 Energy changing between wild-type and mutation

3 Vibrational Entropy Energy changing between wild-type and mutation
